# Health system performance assessment and reforms, Oman

**DOI:** 10.2471/BLT.24.291750

**Published:** 2024-06-04

**Authors:** Taavi Lai, Qasem Al Salmi, Kira Koch, Alaa Hashish, Hamid Ravaghi, Awad Mataria

**Affiliations:** a Fourth View Consulting, Wismari 47a-8, Tallinn 10136, Estonia.; b Ministry of Health, Muscat, Oman.; c Special programme on Primary Health Care, World Health Organization, Geneva, Switzerland.; d World Health Organization Country Office, Muscat, Oman.; e World Health Organization Regional Office for the Eastern Mediterranean, Cairo, Egypt.

## Abstract

**Problem:**

To prioritize key areas of action and investment for the next strategic cycle of national development plans (2026–2031) in Oman, we needed a holistic view of the country’s health system and its main deficiencies and inefficiencies.

**Approach:**

Informed by the World Health Organization framework, our team of seven national health ministry staff and two international experts conducted a rapid health system performance assessment. We used already available data to identify system bottlenecks and their potential root causes, verifying our findings with key informant interviews.

**Local setting:**

Oman’s 4.9 million population is relatively young (average age 28 years) but ageing, with a mounting burden of chronic diseases. While health-care services are free for Omani nationals, more than 1.5 million expatriates rely on out-of-pocket payments for health-care services. Strengthening primary health care, improving the quality of care, providing financial protection, and ensuring that public and private health-care providers operate within the same legal and procedural framework are recognized as key national priorities.

**Relevant changes:**

Our assessment highlighted the need to extend health service coverage to the whole population, strengthen private health-care sector governance, improve health education, increase financial investment, and expand the country’s capacity for data collection and analysis.

**Lessons learnt:**

The assessment framework allowed us to identify areas where information is lacking and use already available data to analyse multiple health outcomes. As well as identifying issues that need to be addressed during the next policy development cycle, our findings have contributed towards the preparation of a more extensive assessment.

## Résumé

**Problème:**

Afin de hiérarchiser les domaines d’action et d’investissement clés pour le prochain cycle stratégique des plans de développement nationaux (2026-2031) à Oman, une vision globale du système de santé du pays et de ses principales faiblesses et inefficacités était nécessaire.

**Approche:**

S’inspirant du cadre de l’Organisation mondiale de la santé, notre équipe, composée de sept membres du personnel du ministère de la Santé et de deux experts internationaux, a procédé à une évaluation rapide de la performance du système de santé. Nous avons utilisé les données déjà disponibles pour identifier les goulets d’étranglement du système et leurs éventuelles causes premières, et nous avons vérifié nos conclusions à l’aide d’entretiens avec des informateurs clés.

**Environnement local:**

La population d’Oman, qui compte 4,9 millions d’habitants, est relativement jeune (l’âge moyen est de 28 ans), mais vieillissante, et le fardeau des maladies chroniques s’alourdit. Alors que les services de santé publique sont gratuits pour les Omanais, plus de 1,5 million d’expatriés paient directement leurs soins de santé. Le renforcement des soins de santé primaires, l’amélioration de la qualité des soins, l’offre d’une protection financière et la garantie que les prestataires de soins de santé publics et privés opèrent dans le même cadre juridique et procédural sont reconnus comme des priorités nationales essentielles.

**Changements significatifs:**

Notre évaluation a mis en évidence la nécessité d’étendre la couverture des services de santé à l’ensemble de la population, de renforcer la gouvernance du secteur des soins de santé privés, d’améliorer l’éducation à la santé, d’accroître les investissements financiers et de développer les capacités du pays en matière de collecte et d’analyse des données.

**Leçons tirées:**

Le cadre d’évaluation nous a permis d’identifier les domaines où des informations font défaut et d’utiliser les données déjà disponibles pour analyser plusieurs résultats en matière de santé. Outre l’identification des questions à traiter lors du prochain cycle d’élaboration des politiques, nos conclusions ont contribué à la préparation d’une évaluation plus approfondie.

## Resumen

**Situación:**

Para priorizar las áreas clave de intervención e inversión para el próximo ciclo estratégico de planes nacionales de desarrollo (2026-2031) en Omán, necesitábamos una visión holística del sistema sanitario del país y de sus principales deficiencias e ineficiencias.

**Enfoque:**

Teniendo en cuenta el marco de la Organización Mundial de la Salud, nuestro equipo, formado por siete funcionarios de los ministerios de sanidad nacionales y dos expertos internacionales, realizó una evaluación rápida del funcionamiento del sistema sanitario. Utilizamos los datos ya disponibles para identificar los obstáculos del sistema y sus posibles causas, y verificamos nuestros resultados con entrevistas a informantes clave.

**Marco regional:**

La población de Omán, de 4,9 millones de habitantes, es relativamente joven (edad media: 28 años) pero está envejeciendo, con una carga creciente de enfermedades crónicas. Mientras que los servicios sanitarios son gratuitos para los ciudadanos omaníes, más de 1,5 millones de expatriados pagan de su bolsillo los servicios sanitarios. El fortalecimiento de la atención primaria, la mejora de la calidad de la atención, la protección financiera y la garantía de que los proveedores de atención sanitaria públicos y privados operan dentro del mismo marco legal y de procedimiento se reconocen como prioridades nacionales clave.

**Cambios importantes:**

Nuestra evaluación destacó la necesidad de ampliar la cobertura de los servicios sanitarios a toda la población, reforzar la gobernanza del sector sanitario privado, mejorar la educación sanitaria, aumentar la inversión financiera y ampliar la capacidad de recopilación y análisis de datos del país.

**Lecciones aprendidas:**

El marco de evaluación nos ha permitido identificar las áreas en las que falta información y utilizar los datos ya disponibles para analizar múltiples resultados sanitarios. Además de identificar cuestiones que deben abordarse durante el próximo ciclo de elaboración de políticas, nuestros resultados han contribuido a la preparación de una evaluación más amplia.

## ملخص

المشكلة حتى نقوم بتحديد الأولوية في مجالات العمل والاستثمار الرئيسية للدورة الاستراتيجية التالية لخطط التنمية الوطنية (2026 إلى 2031) في عُمان، كانت هناك حاجة إلى رؤية شاملة للنظام الصحي في الدولة، فضلاً عن أوجه القصور ونقاط الضعف الرئيسية فيه.

الأسلوب قام فريقنا المكون من سبعة موظفين وطنيين بوزارة الصحة، فضلاً عن اثنين من الخبراء الدوليين، بإجراء تقييم سريع لأداء النظام الصحي، وذلك في ضوء إطار العمل بمنظمة الصحة العالمية. قمنا باستغلال البيانات المتاحة بالفعل لتحديد أزمات النظام وأسبابها الجذرية المحتملة، كما قمنا بالتحقق من النتائج لدينا من خلال المقابلات الشخصية مع مصادر المعلومات الرئيسيين.

المواقع المحلية إن سكان عُمان البالغ عددهم 4.9 مليون نسمة هم من الشباب نسبياً (متوسط العمر 28 عاماً)، ولكنهم يعانون من التدهور الصحي بسبب العبء المتزايد للأمراض المزمنة. وبينما تتوفر خدمات الرعاية الصحية العامة للمواطنين العُمانيين دون مقابل، إلا إن 1.5 مليون وافد يحصلون على خدمات الرعاية الصحية على نفقتهم الخاصة. إن دعم الرعاية الصحية الأولية، وتحسين جودة الرعاية، وتقديم الحماية المالية، وضمان قيام مقدمي الرعاية الصحية في القطاعين العام والخاص بالتشغيل ضمن نفس الإطار القانوني والإجرائي، يتم اعتبارها جميعًا بمثابة أولويات وطنية رئيسية.

التغيّرات ذات الصلة سلّط التقييم الخاص بنا الضوء على الحاجة إلى توسيع نطاق تغطية الخدمات الصحية لتشمل جميع السكان، ودعم إدارة قطاع الرعاية الصحية الخاص، وتحسين الوعي الصحي، وزيادة الاستثمارات المالية، وتوسيع قدرة الدولة على جمع البيانات وتحليلها.

الدروس المستفادة سمح لنا إطار عمل التقييم بتحديد المجالات التي تفتقر إلى المعلومات، وبالتالي استخدام البيانات المتاحة بالفعل لتحليل النتائج الصحية المتعددة. وفضلاً عن تحديد المشكلات التي تحتاج إلى التعامل معها خلال دورة تطوير السياسات التالية، فقد أسهمت النتائج لدينا في إعداد تقييم أكثر شمولاً.

## 摘要

**问题:**

为了确定阿曼在国家发展计划下一个战略周期（2026 至 2031 年）中采取关键行动和选择主要投资领域的优先次序，我们需要全面了解该国的卫生系统及其存在的主要缺陷和效率低下的情况。

**方法:**

在世界卫生组织框架的指导下，我们组成了一支包含 7 名国家卫生部工作人员和 2 名国际专家的团队，并对卫生系统绩效进行了一次简短评估。我们使用已有数据来识别系统瓶颈及其潜在的根本原因，并通过与主要知情者的访谈来验证我们的发现。

**当地状况:**

阿曼 490 万人口的结构相对年轻（平均年龄为 28 岁），但正在老龄化，且慢性病负担日益加重。虽然阿曼公民可以享受免费的医疗保健服务，但该国150 多万外籍人士需要自行承担医疗保健服务相关费用。加强初级卫生保健工作、提高保健服务质量、提供财政保护以及确保公共和私人医疗保健提供者在同一法律和程序框架内提供服务，被认为是国家的主要优先事项。

**相关变化:**

我们的评估结果显示，有必要将卫生服务覆盖范围扩大到全民、加强对私营医疗保健部门的治理、改善健康教育工作、增加财政投入，并提升国家的数据收集和分析能力。

**经验教训:**

评估框架使我们能够识别缺乏信息的领域，并使用已有数据来分析多种健康结果。除了确定在下一个政策制定周期中需要解决的问题外，我们的研究结果还有助于筹备更广泛的评估工作。

## Резюме

**Проблема:**

Чтобы определить приоритетные направления деятельности и инвестиций для следующего стратегического цикла национальных планов развития (2026–2031 гг.) в Омане, требовалось получить целостное представление о системе здравоохранения страны и ее основных недостатках и аспектах неэффективности.

**Подход:**

Опираясь на систему Всемирной организации здравоохранения, команда из семи сотрудников национальных министерств здравоохранения и двух международных экспертов провела экспресс-оценку эффективности системы здравоохранения. Для выявления слабых мест в системе и их возможных коренных причин использовались доступные данные, а полученные результаты проверялись в ходе интервью с ключевыми информаторами.

**Местные условия:**

Население Омана, насчитывающее 4,9 миллиона человек, относительно молодое (средний возраст 28 лет), но стареющее, с растущим бременем хронических заболеваний. Хотя услуги здравоохранения для граждан Омана бесплатны, более чем 1,5 миллиона иностранных граждан, проживающих в этой стране, приходится платить за медицинское обслуживание из собственного кармана. Укрепление первичной медико-санитарной помощи, повышение качества медицинской помощи, обеспечение финансовой защиты, а также обеспечение функционирования государственных и частных медицинских учреждений в единых правовых и процедурных рамках признаны ключевыми национальными приоритетами.

**Осуществленные перемены:**

В ходе оценки была отмечена необходимость расширения охвата населения медицинскими услугами, усиления управления частным сектором здравоохранения, улучшения медицинского образования, увеличения финансовых инвестиций и расширения возможностей страны по сбору и анализу данных.

**Выводы:**

Система оценки позволила выявить недостаток информации и использовать доступные данные для анализа множества результатов мероприятий по охране здоровья. Помимо выявления вопросов, которые необходимо решить в ходе следующего цикла разработки политики, полученные результаты способствовали подготовке более обширной оценки

## Introduction

Health systems are constantly changing; regular wide-ranging assessments of their performance help to identify and adjust priorities,^1^^,^^2^ and inform actions for their robust and resilient transformation. Originally developed by the World Health Organization (WHO)^3^ for this purpose, the health system performance assessment framework becomes an effective tool when combined with policy-cycle and strategic health system development plans.^3^

As part of Oman Vision 2040, the health ministry formulates the design and aims of the country’s health system and submits these for consideration to the Oman 2040 Committee for inclusion in the successive 5-year national development plans.^4^ The Cabinet of Ministers of Oman initiated the development of the next 5-year cycle for the period 2026–2031 in January 2023 by requesting its health ministry to report on the performance of Oman’s health system by April 2023. This performance report enabled the identification of health system investment needs and guided preliminary discussion to develop goals and priorities for 2026–2031.

Here we report on the application and use of the health system performance assessment tool for the first time in Oman, with the specific aim of informing and focusing the planning of reforms in the health sector. 

## Local setting

The population of Oman was 4.9 million in 2023.^5^^,^^6^ The population is relatively young (with an average age of 28 years) but is ageing.^5^^–^^7^ Population growth is slowing^6^^,^^8^ and the burden of noncommunicable diseases (e.g. diabetes) is increasing.^7^ Omani nationals have access to health care from publicly owned health-care service providers. More than 1.5 million expatriates rely on out-of-pocket payments for their health-care needs. Continuous strengthening of primary health care (e.g. by increasing the number of health-care facilities and appropriately trained and funded staff); improving the quality of health care (e.g. developing and updating treatment guidelines); providing financial protection; and ensuring that public and private health-care service providers operate within the same legal and procedural framework are recognized as key national priorities.^9^

## Approach

We chose the health system performance assessment framework published by WHO in 2022^10^ to guide an assessment of the performance of the Omani health system. This WHO framework helps us to review health system functions and subfunctions, understand their interactions, and evaluate their impact on health system intermediate and final outcomes – including the collection and analysis of necessary data – in a systematic manner. Considering the deadline, the health ministry formed a small team to conduct a rapid health system performance assessment using only already available data.

Members of the national assessment team included seven health ministry staff members qualified in a range of individual health system (sub)functions – namely governance, financing, human resources, specialized and primary care service delivery, and pharmaceuticals – and in the integration of these (sub)functions into a national health system. We compiled data describing these individual health system (sub)functions and on intermediate and final outcomes from readily available national^5^ and international databases.^7^^,^^11^^,^^12^ The national assessment team was then joined by two international experts with experience in both the development and application of health system performance assessment frameworks. Our extended team (national and international members) collaborated in the integration of preliminary findings, the identification of data gaps and the collection of such additional data (especially on health system outcomes), the clarification of main findings and the identification of priority areas of action. 

We conducted a stepwise analysis to assess the performance of the health system. Beginning with available data to obtain an overview of results across indicators, we conducted consecutive rounds of analysis where the health outcome findings (e.g. low life expectancy) are verified or their potential factors (e.g. low vaccine coverage, high obesity prevalence or poor accessibility of primary health care) are investigated further with additional data (combinations) or analytical approaches. Such an approach helps to identify the root causes (i.e. health-care practices that can be introduced, extended or improved) of health system bottlenecks (i.e. deficiencies, inefficiencies, restrictions and delays), essential for achieving universal health coverage and improved health security. We then verified our initial identification of such root causes by interviewing key informants, such as health ministry advisors. We conducted 10 such interviews during the assessment process, predominantly with health ministry staff members who were not involved in the assessment process. 

The cost of conducting the rapid health system performance assessment was around 40 000 United States dollars, including material, lodging and the time of national and international expertise. Our health system performance assessment culminated with the presentation of our report^9^ to the health ministry leadership and participation in discussions of our findings. The health ministry then submitted our report, with recommendations for policy action, to the Cabinet of Ministers.

## Relevant changes

Our rapid health system performance assessment identified several changes that should be incorporated within the health system goals and priorities for the next 5-year development plan; several areas of health-care provision and governance should be strengthened, and additional financial investment is required. 

First, health coverage needs to be extended to cover all expatriates so that the entire population is protected against health-related financial risks, ensuring a healthy population and workforce. Second, the health ministry should strengthen the governance of private health-care providers (e.g. by reviewing legislation to ensure health system rules apply to the private sector, and creating enforcement mechanisms to ensure the application of these rules), and ensure that they meet the same quality-of-care and operational standards as the public health-care sector. Third, progress towards services focused on primary health care needs to be invigorated by reviewing and updating the country model of care for more effective and efficient service delivery. Fourth, education to promote health (e.g. nutrition) and the prevention of noncommunicable diseases needs to be improved and better integrated into primary and community health care to reduce the load on secondary and more specialized health care. Fifth, financial investment into areas such as maternal and child health and vaccination should be increased to overcome gradual stagnation of outcomes in this area (e.g. there was only a minor improvement in infant mortality from 10.1 to 9.5 per 1000 live births during 2010–2020)^5^^–^^7^. Sixth, the governance and organization of health-care service providers should be reviewed and updated to improve the quality of care as well as increase the efficiency of resource use. Finally, because gaps in available data need to be addressed before a more extensive performance assessment can be conducted, finance to extend data collection and to ensure data quality and analytical capacity is essential.

## Lessons learnt

We present a summary of the main lessons learnt during this rapid health system performance assessment process in Box 1.

Box 1 **Summary of main lessons learnt**

Use of a rapid health system performance assessment framework in Oman:

- enabled us to obtain a holistic view of health system processes and outcomes, necessary for the identification of inefficiencies, restrictions, delays and gaps in data (e.g. a lack of information about private health-care providers);
- allowed significant insights and benefits despite being conducted using modest resources and data already available; and
- not only identified issues to be considered during the next 5-year policy development cycle in Oman, but also informed the preparation of a more extensive health system performance assessment.

Using such an analytical performance assessment framework enabled us to obtain a holistic view of health system inputs, processes, outputs and outcomes, essential for the identification of bottlenecks in the provision of population-wide health care. For example, the assessment highlighted that the health ministry has little information on the activities and quality of private health-care service providers, demonstrating a lack of effective governance of this sector. Such knowledge deficiencies can mean that problems are not identified and corrective actions are not instigated.

Assessing the performance of a health system creates a high demand for data that few countries could fully meet using routine data collection methods. However, use of the WHO framework for assessing performance allows many common health indicators to provide information about multiple health outcomes. For example, during our rapid assessment we used data describing maternal mortality to gain information on health outcomes, quality of care and the performance of health system functions.

Finally, as well as identifying health-care issues that need to be incorporated within the next 5-year policy development cycle, our rapid assessment has assisted in the preparation for a more extensive health system performance assessment. With the guidance of WHO to ensure impartiality, the health ministry plans to conduct a comprehensive assessment with a larger team that includes representatives from institutions other than the Omani health ministry, and to conduct public consultations on our findings. 

## References

[1] Rajan D, Papanicolas I, Karanikolos M, Koch K, Rohrer-Herold K, Figueras J. Health system performance assessment: a primer for policy-makers. Copenhagen: World Health Organization; 2022. [cited 2024 May 23]. Available from: Available from https://iris.who.int/handle/10665/364198 36800880

[2] Health system performance assessment: reporting and communicating. Practical guide for policy makers. Brussels: European Commission; 2017. Available from: https://health.ec.europa.eu/document/download/165d7fcd-1d09-4ef1-b815-f78a158ef335_en?filename=2017_hspa_reportingcommunicating_en.pdf [cited 2024 May 23].

[3] The world health report 2000. Health systems: improving performance. Geneva: World Health Organization; 2000. Available from: https://www.who.int/publications/i/item/924156198X [cited 2024 May 23].

[4] Oman Vision 2040: Preliminary vision document. Muscat: Oman 2040 Main Committee; 2013. Available from: https://www.national-day-of-oman.info/wp-content/uploads/2020/11/OmanVision2040-Preliminary-Vision-Document.pdf [cited 2024 May 23].

[5] Statistical reports. Muscat: Ministry of Health of Oman; 2024. Available from: https://www.moh.gov.om/en/web/statistics/annual-reports [cited 2024 Apr 7].

[6] Statistics: all indicators. Muscat: National Center for Statistical Information of Sultanate of Oman; 2024. Arabic. Available from: https://www.ncsi.gov.om/Pages/AllIndicators.aspx [cited 2024 May 23].

[7] World Bank open data [internet]. Washington DC: World Bank; 2024. Available from: https://bit.ly/3UIHb5Z [cited 2024 May 23].

[8] Islam MM. Demographic transition in Sultanate of Oman: emerging demographic dividend and challenges. Middle East Fertil Soc J. 2020;25(1):7. 10.1186/s43043-020-00022-7

[9] Rapid health system performance assessment report 2023. Muscat: Ministry of Health of Oman; 2023.

[10] Papanicolas I, Rajan D, Karanikolos M, Soucat A, Figueras J, editors. Health system performance assessment: a framework for policy analysis. Geneva: World Health Organization; 2022. Available from: https://iris.who.int/handle/10665/352686 [cited 2024 May 23]. 37023239

[11] Global health expenditure database [database]. Geneva: World Health Organization; 2024. Available from: https://apps.who.int/nha/database/Select/Indicators/en [cited 2024 May 23].

[12] Eastern Mediterranean health observatory: data repository [database]. Cairo: World Health Organization; 2024. Available from: https://rho.emro.who.int/drs [cited 2024 May 23].

